# ‘Atawhai’: a primary care provider-led response to family violence in Aotearoa New Zealand

**DOI:** 10.1186/s13690-024-01309-1

**Published:** 2024-05-17

**Authors:** Claire Gear, Jane Koziol-McLain, Elizabeth Eppel, Anna Rolleston, Ngareta Timutimu, Hori Ahomiro, Eunice Kelly, Clare Healy, Claire Isham

**Affiliations:** 1 Centre for Interdisciplinary Trauma Research, School of Clinical Sciences, Auckland University of Technology, Auckland, New Zealand; 2 Wellington School of Business and Government, Victoria University of Wellington, Wellington, New Zealand; 3 Centre for Health Manawa Ora, Tauranga, New Zealand; 4 Ngāi te Rangi, Ngāti Ranginui, Tauranga, New Zealand; 5 Tauranga Womens Refuge, Tauranga, New Zealand; 6 Tend South City Medical Centre, Tauranga, New Zealand; 7 Independent forensic practitioner, Christchurch, New Zealand; 8 Western Bay of Plenty Primary Health Organisation, Tauranga, New Zealand

**Keywords:** Participatory research, Primary care, Family violence, Indigenous, Complex adaptive system, Complexity theory, Deliberative dialogue, Complex interventions, Health care

## Abstract

**Background:**

As a key determinant of ill-health, family violence is inadequately responded to within Aotearoa New Zealand health policy and practice. Without adequate system support, health professionals can often be unsure of what to do, or how to help. Developed in response to this system gap, ‘Atawhai’ aims to make it easier for primary care professionals to respond to family violence.

**Methods:**

Underpinned by indigenous Māori customs, Atawhai combines complexity theory and participatory research methodologies to be responsive to the complexity involved in family violence. We worked with 14 primary care professionals across ten whakawhitiwhiti kōrero wānanga (meetings for deliberate dialogue) to identify and develop primary care system pathways and tools for responding to family violence. This paper focuses on the development of Atawhai through wānanga and observation methods. Methods used to capture change will be reported separately.

**Findings:**

Atawhai is a relational response to family violence, focused on developing a network of trusted relationships between health and social care professionals to support safe responses to those accessing care. This study identified four key health system pathways to responsiveness and developed associated tools to support health care responsiveness to family violence. We found the quality of relationships, both among professionals and with those accessing care, coupled with critical reflection on the systems and structures that shape policy and practice are essential in generating change within primary care settings.

**Conclusions:**

Atawhai is a unique health care response to family violence evidenced on empirical knowledge of primary care professionals. Our theoretical lens calls attention to parts of the system often obscured by current health care responses to family violence. Atawhai presents an opportunity to develop a grassroots-informed, long-term response to family violence that evolves in response to needs.

**Table:** 

**Text box 1. Contributions to the literature**
*Research design weaves together indigenous and western science methodologies and methods for equitable outcomes.
*Advances current health system responses to family violence by calling attention to the critical importance of quality relationships between system agents and the initial conditions of complex systems.
*Demonstrates a complex adaptive system approach to translating evidence within health systems.

## Background

*Ehara taku toa i te toa takitahi, engari he toa takitini.* My success is not mine alone, it is the success of the collective (Māori, indigenous people of Aotearoa New Zealand (NZ) whakataukī or proverb, articulated by an Atawhai participant).

Family violence (FV) is a key determinant of ill-health that significantly impacts the health and wellbeing of all family members, past, present and future [1]. Yet internationally, evidencing the effectiveness and sustainability of health care responses to FV has proven challenging, and service provider responses often remain individualistic and transactional [2, 3]. Without adequate system support, health professionals can often be unsure of what to do, or how to help, missing opportunities to provide support [4]. In Aotearoa NZ, there is limited policy, resources, and funding to support the primary care sector to respond to FV [5]. Recognising this gap, this study aimed to identify and develop health system pathways and tools that made it easier for primary care professionals to respond to FV in practice.

This study produced ‘Atawhai’ (to move cautiously with kindness; www.atawhaitia.co.nz), a relational response to FV, focused on building quality network relationships to support the delivery of safe, effective, and sustainable responses to those accessing health care. Developed and led by health care professionals working within primary care settings the ‘Atawhai Kōrero’ (conversations) underpins the response, recognising conversations about FV can occur in many shared moments in time, or wā, within a relationship, underpinned by tika (honesty), pono (truth), and aroha (empathy). Atawhai realises health professionals do not have to ‘fix the problem’ but be someone families and whānau can trust to walk alongside supporting opportunities for change. Care is taken so any kōrero is responsive to, and safe for, families and whānau. This paper reports on the findings of the Atawhai study, answering the research questions (1) What does an effective and sustainable response to family violence look like for primary care, and (2) What influences change in primary care family violence responsiveness? This study provides an example of practical application of complexity theory, where literature remains largely theoretical [6].

### Family violence responsiveness in health care

Health systems have a critical role in FV service delivery, particularly in the primary care setting [7]. Given the consequences of FV on health, it is not surprising that the prevalence of FV among those seeking health care is higher than in the general population [7–9]. While intervention models exist internationally (e.g., RADAR [10], LIVES [11]), there is increasing recognition that standard prescriptive interventions do not reflect the complexity of the problem and are unlikely to generate sustainable solutions [2, 4, 12]. Understanding the relationships between context, violence and ill-health is critical for responding to the needs of those accessing care safely, supporting restoration and healing [13–15].

In Aotearoa NZ, FV is defined as ‘a pattern of behaviour that coerces, controls or harms within the context of a close personal relationship’ [16] (p.10) and is recognised as gender-based, disproportionately affecting women and children [16, 17]. Population-based data estimates nearly two in three Aotearoa NZ women, over two in three Indigenous Māori women, two in five Pacific women and one in three Asian women will experience a form of physical, sexual, psychological, controlling, or economic violence by an intimate partner in their lifetime [18]. Yet, FV is non-discriminatory, also impacting men, older people, disabled, indigenous, migrant and LGBTQIA + communities [16].

Deeply rooted in societal trauma, FV continues to be perpetuated by systems and practices affected by dominant culture and colonial history, with devastating and intergenerational impacts, particularly for Māori [14, 19, 20]. The Waitangi Tribunal [21] has found significant breaches of Te Tiriti o Waitangi (the Māori language version of the written agreement between the British Crown and more than 500 Māori chiefs signed in 1840), which, historically and today, have resulted in broad inequities in Māori health outcomes, reinforced by chronic underfunding of Māori health services [22, 23]. Understanding how colonialist and other system structures shape health care responses to FV, and therefore the agency and choices whānau and families have, is critical to disrupting patterns of violence for Māori and all populations in Aotearoa NZ [19, 24].

Primary care is consistently identified in health strategies and policy as a priority setting where disproportionate numbers of people impacted by FV present [5, 7, 25]. Yet in Aotearoa NZ, the sector continues to be underutilised in the cross-government work to reduce FV with limited guidance and resourcing, generating ad hoc practice [4, 5]. Launched in December 2021, Te Aorerekura is the National Strategy and Action Plan to eliminate family and sexual violence [16, 26]. Capability frameworks were launched in May 2022 to build government and non-government workforce capability in responding to FV [27, 28]. Health care is considered a ‘generalist workforce’, who must know how to respond safely and effectively to FV [29]. The strategy includes the existing Te Whatu Ora (Health New Zealand) Violence Intervention Programme (VIP), and primary care clinician workforce training for recognising and responding to sexual assault and/or non-fatal strangulation provided by Medical Sexual Assault Clinicians Aotearoa (MEDSAC) as work already under way in the health care sector. Beginning in 2004, VIP has established significant system infrastructure within hospitals and selected community settings over time to support intimate partner violence and child abuse and neglect identification, assessment, and referral. However, longitudinal evaluation data evidences low assessment and disclosure rates [30]. Primary care professionals argue the VIP intervention guidelines are inappropriate for the primary care setting and consider response autonomy important, highly valuing a local response, for the local context, supported by local relationships [31–33]. It is therefore critical to engage and empower primary care professionals in developing a response to FV that aligns with the motivations and concerns of their settings and contexts [34]. This study convened a series of whakawhitiwhiti kōrero wānanga (meetings for deliberate dialogue), to work with primary care professionals in the development of a FV response suited to such diverse contexts.

## Methodology

Grounded in tikanga Māori (indigenous ways of being, relating and doing), this study combined complexity theory [35] and participatory research [36, 37] methodologies to create a theoretical lens guiding study development, conduct, analysis, and impact. Methods included ten participant one-day whakawhitiwhiti kōrero wānanga (meetings for deliberative dialogue), Kaihopu Kōrero observation (conversation catchers), social network analysis (SNA) and pre/post readiness surveys. This paper focuses on the development of Atawhai through whakawhitiwhiti kōrero wānanga and Kaihopu Kōrero methods and discusses implications for policy and practice. SNA and readiness survey findings are reported separately.

### Reflexivity

The premise of this study originates from over a decade of research seeking to improve responsiveness to FV within the Aotearoa NZ primary care sector [5, 31, 38–40]. Guided by our theoretical lens, we hypothesise effective and sustainable responses to FV are emergent from the interaction between the health professional and person(s) accessing care [38], leading to our focus on working with primary care professionals in understanding one part of this system interaction. The study is evidenced on the understanding that FV is (a) a key determinant of ill-health, (2) a complex problem, (3) a profound system gap, (4) an urgent issue, and that (5) primary care is a window of opportunity to make a difference [3, 7, 25]. The development and conduct of this study is grounded in research team relationships which have developed over long periods of time. Given the importance of this research to Māori, our team is privileged to be guided by two kaitiaki (protector) who protect the mātauranga (knowledge) and tikanga Māori involved in the research. Additionally, five investigators hold whakapapa (tribal relationships) to the research location. Our team includes highly skilled researchers and leaders in the fields of qualitative research methods, violence against women, primary care, Māori health research, complexity theory and specialist community FV services. Four investigators work within primary care service delivery settings.

### Methods

This study was conducted in the Bay of Plenty (BOP), a region in the North Island of Aotearoa NZ. Prior to health reforms in 2023, capitation funding was distributed via District Health Boards (DHBs), responsible for planning and delivering health care services for their regional population. DHBs contracted primary care via service agreements with Primary Health Organisations (PHOs), responsible for contracting service delivery providers, largely general practices [23]. The BOP DHB served a population of approximately 225 thousand people; had a higher proportion of Māori compared to the national average (25% vs. 16%); and included three PHOs [41].

This study was approved by the Auckland University of Technology Ethics Committee (21/31). Fourteen participants (ten women, four men) were recruited via word of mouth (facilitated by research network relationships), a study website (www.atawhaitia.co.nz) and advertisements within local health care newsletters. The lead researcher met (online or in person) with potential participants to discuss study aims and expectations and answer queries or concerns. Following this discussion, a formal invitation with information sheet and consent form were emailed. Information sheets were also made available via the study website. We invited participant organisations to endorse their participation in the study via informed consent. Facilitated by participants, organisational consent (provided by senior management) aimed to provide managerial support for participants to attend wānanga and recognition as a participating organisation in project communications and publications. Organisations and/or participants were offered reimbursement of costs incurred by participating including time, travel, accommodation, and sundry costs.

Recruited participants included five tauiwi (non-Māori) and nine Māori, including general practitioners (two), a nurse practitioner (one) and a practice nurse (one), social workers (seven) and managers (three). Four participants were employed within general practice clinical settings and ten within Māori health organisations. Retention fluctuated over the study period, with participants navigating shifting employment, roles, managers, and capacity. Two participants withdrew following the first wānanga due to conflict in worldviews. One participant withdrew following the seventh wānanga due to a process issue preventing reimbursement for participation time. Role reassignment meant three participants were not supported by organisation management to continue to attend wānanga, though they remained connected to the study via communications. A core group of eight remained active participants throughout the study. Manaakitanga (the process of showing respect, generosity, and care for others), and the developing shared sense of commitment, connection and learning amongst the participants and research team was key to retention. The COVID-19 pandemic significantly impacted health care service provision, with many participants reporting their availability and capacity to participate in the project was stretched. During the data collection period there were two periods in which the whole country was mandated to isolate at home (25 March 2020–27 April 2020; 17 August 2021–31 August 2021) [42]. The COVID-19 Protection Framework (Traffic Lights) was in effect from 2 December 2021 to 12 September 2022 [43].

### Data collection

*Whakawhitiwhiti kōrero wānanga, deliberative dialogue workshops.*

Atawhai emerged from ten, one-day, in person whakawhitiwhiti kōrero wānanga over 22-months (4 August 2021–29 June 2023). Aligned with deliberative dialogue methods [44–46], wānanga bring people together to kōrero about a common kaupapa (topic) and learn from one another, advancing knowledge [44–46]. The Atawhai wānanga supported participants to draw on their contextual knowledge and practice to critically reflect on *what* information is required for responsiveness to FV in primary care and *how* it may be integrated into practice. Each wānanga was planned and conducted by facilitators, Kaihopu Kōrero (see below) and kaitiaki, responsive to participant needs. Additional research team members attended wānanga as needed (e.g., for knowledge translation). The agenda for each wānanga included karakia (prayer), mihi whakatau (welcome), whakawhanaungatanga (relating to others), and innovative activities to build upon what participants identified as needed. Activity highlights included being welcomed to a local marae (meeting house) by iwi (tribe), being gifted our own waiata (song) by kuia (elder), producing video vignettes on the value of the research, and the Minister for the Elimination of Family Violence and Sexual Violence attending Wānanga Six [47]. Table 1 lists the general topics discussed at each wānanga.

**Table 1:** Atawhai wānanga discussion topics

Wānanga	Discussion topics
One	• Social connection • Individual pathways to family violence responsiveness • Uncertainty and doubt in practice • Understanding local history, whenua (land), whakapapa (genealogy), culture and impact of colonisation and racism
Two	• Critiquing health care responses to family violence • Identifying existing participant knowledge and strengths • Generating meaningful connections across networks
Three	• Establishing a kawa (protocol) for group work • Taking care of yourself first • Identifying values for connecting and communicating
Four	• Identifying the purpose of an Atawhai network and how it is different to others • Discussing benefits, challenges, and possibilities of a network • What a perfect scenario of family violence responsiveness looks like
Five	• Reconnecting to motivations to respond to family violence • Validating an Atawhai Common Language • Sharing practice ‘gems’
Six	• Considering what is needed to make practitioners and organisations safe • Advocating Atawhai with government leadership
Seven	• Findings from Te Ao Māori (Māori worldview) • Identifying intellectual property and Mātauranga Māori protections • Identifying opportunities to promote Atawhai
Eight	• Revisiting tangata whenua (local indigenous Māori) at marae (meeting place) • Welcoming new network members • Building Network needs, peer support and supervision
Nine	• Validating preliminary overall research findings • Considering knowledge gaps • Discussing what Atawhai looks like post-research
Ten	• Building the Atawhai Network • Planning regional network wānanga

Participant connection and learning occurred both inside and outside of the wānanga. For example, regular opportunities to connect online or in-person were facilitated to nurture relationships amongst the participants and research team. Participants also held conversations with colleagues about Atawhai, analysing what was learnt and bringing that learning back to wānanga. Data saturation was considered achieved at Wānanga Seven where participants agreed on what they had identified and developed, and discussions turned to advocacy, influence, and implementation. Participant attendance at wānanga fluctuated depending on participant availability, capacity, illness, tangihanga (funeral) etc. The facilitated opportunities to connect in between wānanga were key to keeping participants and the research team connected. On average, six participants attended each wānanga, eight or more participants attended in wānanga one and two. Wānanga Eight, Nine and Ten included new interested people (Atawhai Network members) in addition to participants. All people who engaged in the research were provided a list of FV and mental health service providers options as well an offer to debrief with research team members at any time.

### Kaihopu Kōrero

During the wānanga, two senior research team members served as Kaihopu Kōrero (conversation catchers), modifying the UN Special Rapporteur role [39, 48]. Kaihopu Kōrero were responsible for (1) ensuring discussions were of value in addressing our research aims informed by complexity theory and tikanga Māori, (2) capturing participant interactions and decisions and challenging thinking, (3) reporting observations and decisions to participants for validation, and (4) informing subsequent directions and diffusion strategies. Utilising Kaihopu Kōrero reflections, facilitators presented previous learning and decisions to participants for feedback at strategic points during the wānanga. Participants were encouraged to share their reflections on the previous wānanga during the whakawhanaungatanga phase. Following each wānanga, the research team debriefed and reviewed the Kaihopu Korero notes to create a one-page summary that was provided to participants. Designed as an internal knowledge translation mechanism to keep participants and the research team connected, each summary included key themes discussed at the wānanga, participant quotes and photos, knowledge needs and next steps. Learning and next steps were discussed with the wider research team at monthly meetings.

### Analysis

Our theoretical lens calls attention to how knowledge is emergent from continuous negotiation between multiple ways of knowing, inclusive of the practices, cultures and contexts involved in health care provision [49]. For example, understanding ‘what is the problem’ evolves in response to new knowledge. As such, analysis was adaptive and responsive to real-time findings as participants and the research team interacted and learned from one another. Utilising Kaihopu Kōrero data, real-time analysis involved mapping patterns of interaction and co-created pathways to responsiveness as they emerged during learning. It was a process of weaving together many data elements over time, allowing for new understanding to emerge for consideration by participants at subsequent wānanga. For example, the *Atawhai Common Language* (see findings) is a weave of the collective understanding of the problem of FV developed at wānanga two and the fundamental principles and values for connecting and communicating with whānau and families identified at wānanga three. Similarly, initial discussions with participants on growing meaningful relationships to support practice led to exploration of what an informal alliance could look like, that led to the realisation of the Atawhai Network (see findings).

## Findings

Our primary research question asked, ‘What does an effective and sustainable response to family violence look like for primary care?’ This study found it is “having a network of trusted relationships between clinicians and kaimahi (community service providers) who share skillsets and information to support safe, relational responses to whānau and families, responsive to complex needs over time.”

Participants trusted in their mana (leadership), rangatiratanga (collective self-determination) and expertise, to deliver and advocate for safe FV support and interventions for whānau and families within their respective communities. What this looks like in practice is demonstrated by the *Atawhai Kōrero (conversation), Atawhai Network*, and *Atawhai Common Language*. This is supported by four *Pathways to Responsiveness.* To view these resources, visit www.atawhaitia.co.nz.

The Atawhai response to FV is encapsulated within the *Atawhai Kōrero.* It recognises that:

Kōrero [conversations] about family violence can be many shared moments in time, or wā (time), within a relationship, underpinned by tika (honesty), pono (truth), and aroha (empathy). Atawhai realises that as practitioners, we do not have to ‘fix the problem’ but be someone whānau and families can trust to walk alongside supporting opportunities for change. Care is always taken so any kōrero is responsive to, and safe for, whānau and families.

Responsive to participant learning, the *Atawhai Network* was developed to bring together primary care professionals who are dedicated to preventing FV. Underpinned by the *Atawhai Tikanga*, this health-sector-led network connects health care professionals and organisations with other providers, information, and tools to safely journey with whānau and families in their experience of FV. Atawhai relies on developing relationships across settings to share and learn from one another and translate knowledge between different health care disciplines and contexts. Members of the Atawhai Network gain confidence in knowing what to do and how to help, develop trusted relationships with local referral services and can share challenges with like-minded people. As described by a participant, the Network provides a supportive environment to build confidence and assist with the uncertainty involved in responding to complex care needs. The Atawhai Kōrero works not as a ‘tick-box’, but a ‘touchstone’ to refer to when working in an isolated consultation. Although not a quick fix, the Atawhai Network is a long-term health care response to FV that continually evolves in response to need.

Guided by Thomas and McDonagh [50] and developed from the collective understanding developed between Wānanga Two and Three, the *Atawhai Common Language* (Table 2) was validated by participants at Wānanga Five. The Atawhai Common Language articulates a shared language and behaviour for an effective network response, including a shared understanding of the value of participating. As described by a participant, Atawhai presents an opportunity for practitioners and organisations to *recognise the responsibility* primary care has to respond to FV as a key determinant of ill-health and *realise the unique value* of the consultation space for safe kōrero about FV. The Atawhai *Pathways to Responsiveness* recognise that many small changes in patterns of interaction over time will lead to system change. When enacted, these four health care system pathways will support sustainable responsiveness to FV: (1) Establishing FV as a key determinant of ill-health, (2) Connecting medical and community service provision, (3) Advocating for clinical and cultural supervision for practitioners, and (4) Tuituia: Connecting to information and support.

**Table 2:** Atawhai Common Language*

**Why** The impacts of family violence **seriously affect the health and wellbeing** of past, current, and future generations. Family violence is **complex**, there is no simple solution. Sometimes it can be hard to know what to do or how to help. Atawhai **connects** primary care providers to **local** knowledge and resources to **prevent** violence within the community.	**How** The Atawhai Kōrero creates space in a moment in time to **be present**, to **pause** and to **breathe**. We engage with care-seekers **authentically** and **openly**, with compassion and respect. Atawhai generates **confidence** in knowing how to help and trust in local services and people. We share and learn from our experiences to be helpful for those seeking care.	**What** Atawhai is a **korowai** (cloak) of care that supports the journey of providers, whānau and families. We are part of a team that **values and looks after one another** in this work. Atawhai is a kete (basket) of local information, people and tools that enable us to journey **safely** with whānau and family. Atawhai is a **call to action** to support primary care providers in responding to family violence through policy and practice.

*Bolded words are Atawhai values identified by participants in wānanga three.

Our secondary research question asked, ‘What influences change in primary care family violence responsiveness?’ This study found two key influences: (a) relationships and (b) critical reflection. Within primary care, relationships between professionals, and between a professional and person(s) accessing care, are key to influencing change. We encouraged trusted relationships to occur through regular in-person whakawhanaungatanga (relating to others), kai (food) and kōrero (conversation). Across the wānanga, Atawhai generated trusted relationships between practitioners enabling a safe space for difficult, sensitive, inspiring, and hopeful conversations about FV to occur. Participants noted opportunities for learning from diverse relationships, including whānau, families, other colleagues, and their own experience. Over time, the research team, and participants (both collectively and separately) were able to generate ‘Āhuru Mōwai’, safe spaces for conversations about FV. Uniquely, Atawhai was also able to develop trusted relationships between the often-conflicting worldviews of western clinical practice and Māori health. As captured by Kaihopu Kōrero, participants said:

“[There is a] great sense of possibility and openness and potential. A re-engagement of medicine with te ao Māori. The big thing is about trust. To regain trust where trust has been lost. To use the wairua [spirit] of Atawhai to build and regain trust. Honours both sides, Māori and medical. A dignified way of entering into a relationship” (Participant, wānanga seven).

“At the beginning [I was] feeling disheartened and at a loss to know how to help, [I] came to the first wānanga looking for solutions. [I’m] not feeling so powerless now, knowing there are other people who strongly feel this is something important. We don’t need to fix it, but we are all here to support each other” (Participant, wānanga seven).

“After the first wānanga I was scared and reluctant to come back because I thought all of you are doing such good work – but now having shared that kōrero with you about what we are trying to do in Atawhai and how you are approaching the challenges, I feel so good and I know that I could call on you for help” (Participant, wānanga two).

The second key influence for change was generating opportunities for primary care professionals to have time and space to critically reflect on how personal, organisational, political, and societal system structures shape responses to FV. Participant diversity allowed learning from different perspectives, and these reflections often initiated a transformation in how participants thought about a FV response that then influenced how they practiced. As captured by Kaihopu Kōrero, participants expressed the following:

“You come in [to the wānanga] with one intent and come out with something different and it is evolving all the time” (Participant, wānanga five).

“[We’ve] built up a lot of shared knowledge that has been distilled into many different resources. New knowledge grounded in practice that can inform policy […] I feel that is what a lot of practitioners are hungry for” (Participant, wānanga seven).

In another example, community service providers became aware that clinical providers did not routinely receive formal supervision, which was viewed as a serious lack of practice support. “Supervision” in this context, refers to a process of professional learning and development that enables individuals to reflect on and develop their knowledge, skills, and competence, through agreed and regular support with another professional. In lieu of this support, a peer support group in one primary care clinic setting was set up to provide a safe space for clinical providers to share the challenges they face in practice. As captured by Kaihopu Kōrero, participants reflected:

“Kaimahi [workers] on the ground need to be well to do this job, therefore manaaki in the form of supervision and peer support is necessary” (Participant, wānanga eight).

“Young doctors […] were really shell-shocked by managing FV. There is nothing in the curriculum about violence, so they are feeling not prepared to manage [FV] especially because it is confronting. There’s a discrepancy between the view of students that it’s not in the curriculum and the university who say it is. But it’s not enough to help young docs feel comfortable. It’s not landing for students” (Participant, wānanga seven).

Participants strongly argued that to effectively address FV one had to “Know first who you are and where you come from” to “Be authentic” in the relationship with someone accessing care. These values were articulated within Atawhai ‘Gems’, personal maxims that helped practitioners converse about FV in practice (see Table 3 and visit www.atawhaitia.co.nz).

**Table 3:** Example Atawhai ‘Gems’

You don’t have to have a solution, sometimes listening is all that’s needed.
What would I say to me?
Don’t feel bad if you think you might not have done or said the ‘right thing’ – you can learn from that and do something different next time.

## Discussion

Family violence is a key determinant of ill-health inadequately responded to within health systems internationally [3, 51]. The World Health Organisation advises health systems to establish comprehensive system infrastructure that supports a ‘ women-centred, first-line’ response to intimate partner violence [7]. There is also growing recognition that standard prescriptive interventions do not reflect the complexity of the problem generating design of more adaptive and diverse methodological frameworks [38]. The innovative research design of this study pushes against methodological boundaries, operating as a complex adaptive system itself [52], shifting and changing in response to what is observed and learned during the research. This study developed ‘Atawhai’, a unique relational response that aims to make it easier for the primary care sector to respond to FV in Aotearoa NZ. We discuss two key learnings from this study that influence change (1) providing opportunity for critical reflection on the systems and structures shaping policy and practice, and (2) valuing trusted relationships for practitioners and those accessing care.

### Critical reflection: why do we respond the way we do?

The UK Kings Fund describes transformational change in health care as requiring “a fundamental rethink to find new and better solutions. It requires a shift in the power balance within relationships, in mindsets and in ways of working, at every level of a system” [53] (p.84). Our findings show to generating change in responsiveness to FV requires greater attention to the initial conditions of complex adaptive systems (CAS). Within FV, there is a myriad of personal, organisational, political, and societal elements constantly interacting and shaping how the ‘problem’ is viewed (epistemology), and consequently, how we design interventions (methodology). The intervention design has implications for how responsiveness to FV is articulated within policy and demonstrated in practice (methods). However, what is often lacking is critical reflection on the systems and structures underpinning individual and collective stances on the problem (e.g., biomedicine, gender, equity, racism). These influences shape how we design responses to FV and therefore how responsive we are to FV in practice [51, 54–56]. Atawhai found providing health professionals the opportunity to critically reflect on assumptions underpinning personal and organisational service delivery can initiate transformative change in the way a response is thought about. This finding builds on the ‘Triple R Pathway’ [4], our CAS approach demonstrating the self-organisation of FV responsiveness within health care. Having space and time to think and engage in conversation with peers influences the ‘Respond stance’, altering the pathway toward ‘Responsiveness’ in practice [4, 34].

Complexity theory helps to see that interventions do not exist in isolation and are dependent on the context they occur within [34, 57]. System and structural elements that exacerbate FV and shape the choices people have for change (such as housing, poverty, patriarchy, colonialism) are well known and addressed with approaches such as trauma and violence-informed care [13], or ecological models [1] that call attention to relationships with social determinants of health. Similarly, indigenous-centred approaches to FV service delivery recognise the impacts of colonisation, racism, and collective trauma over time [14, 19, 58]. The importance of critical reflection was made apparent as participants made sense of the patterns of interaction between systems and structures and how these were shaping their practice. Further, through conversation, participants could understand and empathise with *others’* motivations and perspectives as well as the contextual constraints, expectations, pressures and uncertainties they faced, providing a wider view of the complex systems they each operated within [34]. This learning generated a humility amongst the participants and insights, recognising they are one part of a much larger system.

Critical reflection adds to the concept of being ‘ready’ to respond [59], requiring deep self-exploration of personal motivations to address FV. This learning could also take place within a collective (e.g., primary health care organisation), reflecting on organisational identity, values, and principles in relation to responding to FV and congruence with current practice. At a health system level, critical reflection on how the current public health approach to FV influences the development of interventions and resources is needed [55]. For example, screening, or routine enquiry, can obscure patterns of interaction with the systems and structures that exacerbate FV and entrapment, reducing understanding of the complexity involved [33]. The Atawhai complex adaptive system approach to FV connects problem to context, prompting reflection on why we respond the way we do. Our findings align with ‘simple rules’ for translating evidence in complex systems; ‘acting scientifically and pragmatically’, ‘embracing complexity’, and ‘engaging and empowering’ those affected by the desired change [34].

### Relationships: creating safe spaces for conversation and learning

Utilising complexity theory, Atawhai was premised on the hypothesis that sustainable responses to FV are emergent from the interaction between the clinician and person accessing care [38]. Understanding sustainability as an emergent phenomenon means the quality of relationships between system agents is critical to success [60, 61]. Atawhai demonstrates the critical importance of trusted relationships for addressing FV, which is often taken for granted, or undervalued. The Atawhai Kōrero guides developing genuine connection and understanding through unstructured conversation with those accessing care. Following the hypothesis, a mutual understanding of circumstances and a way forward emerges through conversation. Each person (professional and care-seeker) in the relationship learns from the kōrero which changes the way they act. Through these interactions, change occurs. However, given the non-linear nature of conversation and learning, there is uncertainty about whether change will be considered positive or negative. From this CAS perspective, as Jordan, Lanham et al. [61], say, “It is not possible to first learn about an intervention, then plan the intervention, and then implement the plan. Rather, individuals and collectives must learn as they act, and they must act in order to learn” (p.6). This approach is substantially different from the current public health method of identifying, assessing, and referring FV, which is often transactional, in response to a single event, and delivered at a single point in time [30, 33]. Atawhai allows for kōrero as often as needed, aligning with primary care aims of long-term relationships with families and whānau.

Atawhai also highlights the critical importance of building trusted relationships amongst diverse primary care professionals to address FV. Building trusted relationships amongst professionals is often cited as necessary for better FV service provision, yet how to do so is often unclear. Getting to ‘Āhuru Mōwai’, our safe space as a group, was a journey that took time for trust to be established. Trust emerged from participants and the research team learning about one another (whakawhanaungatanga), describing their role, constraints, access to resources, and worldviews. Once trust was established, participants felt safe to engage in critical reflection, learning and sensemaking with one another [60]. Time is often cited as a barrier to delivering services or attending training within general practice due to the increasing complexity of care, workload expectations and administrative burden [62]. Trust takes time to occur and is mediated by many systemic influences, such as an expectation to achieve a trusted relationship within a 10–15-minute consultation. The Atawhai Kōrero advocates for many shared moments in time to occur, for the primary care profession to be someone that will walk alongside them in their journey. Having effective system supports such as referral relationships can work toward clinician capability in addressing concerns [63].

From a CAS perspective, FV interventions that focus on increasing the quality of relationships amongst diverse primary care professionals are likely to see the emergence of sustainability [60]. Based on relationships, Atawhai is an adaptive and reflexive approach, responsive to the uniqueness of local systems. It acts as a ‘boundary vehicle’ [64], providing a set of principles and values that can be reinterpreted over time by individuals and collectives based on their critical reflection and learning. We hypothesise Atawhai will generate synergies within existing systems, rather than acting as a standalone intervention, for example, building capability through supervision for general practitioners. Our learning offers three empirical rules to improve responsiveness to FV in primary care: (1) know yourself and the world in which you usually operate to realise and understand the system boundaries you reinforce every day through language and behaviours, (2) create, learn and adopt a common language that can work across worlds and continue to grow and enrich that language through conversation and use, and (3) create ‘boundary vehicles’ that work to bridge the boundaries of different worlds.

### Strengths and limitations

This study demonstrates a complex adaptive system approach to translating evidence within health systems. It successfully weaves together indigenous and western science methodologies and methods to produce the equitable outcomes of Atawhai. The clear theoretical position directed our attention to patterns of relationships from both Te Ao Māori and complexity theory worldviews. Findings highlighted a need to critically reflect on the underpinning systems and structures that shape the design of responses to FV. Early in the study, we presented participants with an evidence base of FV as (1) a key determinant of ill-health, (2) a complex problem, (3) a profound system gap, (4) an urgent issue, and (5) primary care as a window of opportunity to make a difference. While participants critiqued this evidence themselves, on reflection we understand this to have underpinned the development of Atawhai. Participant recruitment was directed toward those associated with a general practice or Māori health organisation which limited engagement with other primary care settings such as those providing physiotherapy or midwifery services. Recruitment was limited to one geographical region of Aotearoa NZ and subject to selection bias (i.e., those interested in FV agreed to participate and more participants were recruited from Māori health organisations than clinical practices). A key limitation to the scale-up and spread of Atawhai was the engagement of the decision makers within participant organisations. While participants saw the value and benefit of Atawhai, it was more difficult to communicate this to those ‘outside’ of the study. Engaging decision-makers along the way is critical to response development, a key insight for future participatory research. The sustainability of the Network will depend on leadership and funding post-research. Finally, we focused on making it easier for primary care professionals to respond to FV. Future research will engage with whānau and families to explore what matters to them when accessing services, capture evidence of service delivery change (positive and negative), and monitor for unintended consequences over time.

## Conclusions

Atawhai is a unique health care response to FV evidenced on empirical knowledge of primary care professionals. It advances current thinking on health system responses to FV by calling attention to two system parts (relationships and critical reflection) often not considered in current responses. Transformative change relies on the critical need to reflect on the systems and structures that shape responses to FV. Sustainability relies on the quality of system relationships in creating safe spaces to talk about FV. Generating change in complex systems takes time. Not a ‘quick fix’, Atawhai presents a grassroots approach to developing a sustainable long-term response to FV that evolves in response to needs. Most importantly, Atawhai aims to prioritise developing trust, so that whānau and families feel comfortable and heard amidst a plethora of complex and multiple traumatic experiences and needs.

## Data Availability

Study website (www.atawhaitia.co.nz) provides anonymised collective findings.

## Abbreviations

FV: Family Violence
NZ: New Zealand
VIP: Violence Intervention Programme
CAS: Complex adaptive system
BOP: Bay of Plenty
DHB: District Health Board
PHO: Primary Health Organisation
